# Bridging lipid metabolism and mitochondrial genome maintenance

**DOI:** 10.1016/j.jbc.2024.107498

**Published:** 2024-06-27

**Authors:** Casadora Boone, Samantha C. Lewis

**Affiliations:** 1 Department of Nutritional Sciences and Toxicology, University of California, Berkeley, California, USA; 2 Department of Molecular and Cell Biology, University of California, Berkeley, California, USA

**Keywords:** mitochondria, lipid metabolism, mitochondrial DNA (mtDNA), lipotoxicity, mitochondrial metabolism

## Abstract

Mitochondria are the nexus of cellular energy metabolism and major signaling hubs that integrate information from within and without the cell to implement cell function. Mitochondria harbor a distinct polyploid genome, mitochondrial DNA (mtDNA), that encodes respiratory chain components required for energy production. MtDNA mutation and depletion have been linked to obesity and metabolic syndrome in humans. At the cellular and subcellular levels, mtDNA synthesis is coordinated by membrane contact sites implicated in lipid transfer from the endoplasmic reticulum, tying genome maintenance to lipid storage and homeostasis. Here, we examine the relationship between mtDNA and lipid trafficking, the influence of lipotoxicity on mtDNA integrity, and how lipid metabolism may be disrupted in primary mtDNA disease.

Mitochondria are double membrane-bound, energy producing organelles that arose as a result of an ancient endosymbiosis event (1). Over millennia, mitochondria have co-opted host cell resources, and in the process, host and endosymbiont metabolism have become inextricably linked (1). Integration of metabolic pathways at mitochondria relies on their highly organized ultrastructure, consisting of an outer mitochondrial membrane (OMM) porous to metabolites but not to proteins and a structured inner mitochondrial membrane (IMM), separated by an intermembrane space. Within the matrix, mitochondria contain a small polyploid genome, packaged into nucleo-protein structures termed ‘mitochondrial nucleoids’ and distributed throughout dynamic networks (2). Nucleoids are thought to closely associate with the inner membrane, are limited in their diffusion by the IMM cristae, and are segregated to daughter organelles in a regulated manner by mitochondrial division (3). The thirteen protein-coding genes in the human mitochondrial genome are subunits of respiratory chain complexes that execute oxidative phosphorylation (OXPHOS) for energy production in the form of adenosine triphosphate (ATP). MtDNA quality control and euploidy are critical to cellular energy production (Fig. 1*B*).

**Figure 1:**
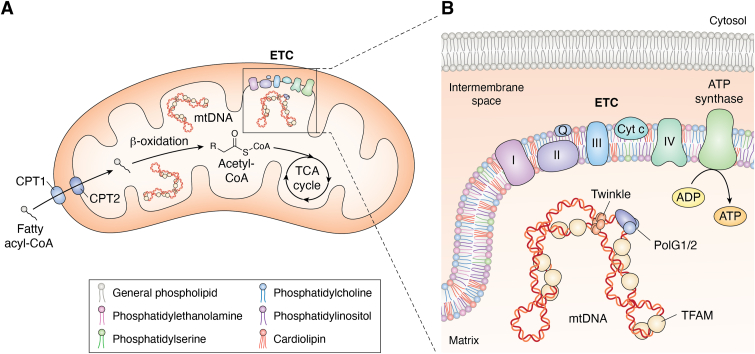
**Mitochondrial ultrastructure and genome localization.***A*, Mitochondrial β-oxidation and respiration are displayed. Cytosolic fatty acyl-CoA molecules are converted into acylcarnitine by CPT1 at the outer mitochondrial membrane (OMM) and reconverted into acyl-CoA by CPT2 at the inner mitochondrial membrane (IMM) upon entering the mitochondrial matrix. Acyl-CoA is broken down into acetyl-CoA that can be channeled into the TCA cycle for respiration. *B*, The OMM and IMM are distinct in their structures and displayed are phospholipids that make up the IMM, with cardiolipin being unique to the IMM. The mitochondrial genome (mtDNA) is considered to be localized near the IMM, which is also the location of electron transport chain complexes (complexes I, II, III, IV, coenzyme Q, cytochrome c, and ATP synthase), whose functions result in ATP production. mtDNA is compacted into a protein–nucleic acid complex called the nucleoid, with TFAM being necessary for its compaction. Mitochondrial replication is facilitated by nuclear-encoded PolG1 bound to processivity factor PolG2, and Twinkle is the helicase that unwinds mtDNA for replication. ATP, adenosine triphosphate; CPT1, carnitine palmitoyltransferase 1; CPT2, carnitine palmitoyltransferase 2; Cyt C, Cytochrome C; ETC, electron transport chain; PolG1/2, polymerase gamma 1 and 2; Q, coenzyme Q; TCA, Tricarboxylic acid cycle; TFAM, mitochondrial transcription factor A.

Over two thousand proteins are required for mitochondrial biogenesis *in toto*, thus the vast majority of mitochondrial proteins are encoded in the nuclear genome and imported into mitochondria by dedicated, membrane-associated complexes at the outer membrane. Unlike organelles such as cilia or the mitotic spindle, mitochondria cannot be built *de novo* (4, 5). Mitobiogenesis depends on the import of proteins, lipids, and other metabolites to extend the pre-existing network. Thus, mitochondrial mass, form, and function are highly dependent on cellular resources and metabolic signaling (6). This includes lipids for mitochondrial membrane growth provided by diet or *de novo* synthesis in animals.

Mitochondria play key roles in lipid oxidation and synthesis, and there is growing evidence that physical interactions between mitochondria and other organelles sustain the context-dependent balance necessary for cellular lipid homeostasis, as reviewed elsewhere (4, 5). Dysregulation of lipid homeostasis has damaging effects on mitochondrial form and function, including endoplasmic reticulum (ER) membrane contacts and fission factors that play roles in the mtDNA synthesis pathway. Here, we discuss roles of mitochondria in lipid metabolism and highlight how these pathways are tied to mtDNA maintenance.

### Mitochondrial networks balance lipid oxidation and synthesis to affect homeostasis

Mitochondria can metabolize multiple substrates to power ATP production, including glucose and fatty acids. Glucose, the preferred fuel source, is ultimately converted into acetyl-CoA molecules that channel into the citric acid (TCA) cycle. The TCA cycle provides metabolites to serve as electron carriers that donate electrons to IMM-resident electron transport chain (ETC) complexes with mtDNA-encoded subunits. Four protein complexes at the IMM pump protons to acidify the intermembrane space, generating a gradient that protons are permitted to run down to power ATP synthase complex, driving the phosphorylation of adenosine diphosphate (Fig. 1, *A* and *B*). However, when glucose availability is low, such as during starvation, lipids provide an alternative fuel source for ATP production. The breakdown of acetyl-CoA supplies the substrate needed for respiration to continue to meet cellular energy demands.

Mitochondria conduct beta-oxidation of short (≤6 carbons), medium (8–14 carbons), and long-chain fatty acids (15–21 carbons) and thus play a crucial role in cellular lipid catabolism. Indeed, while peroxisomes partially catabolize long chain and very long chain fatty acids (≥22 carbons), their oxidation is completed within mitochondria. Upon entry into the cell, fatty acids may be trafficked directly to specialized lipid droplet (LD) organelles for neutralization and storage, to the ER for phospholipid synthesis and LD biogenesis, or to mitochondria. Mitochondria uptake these lipids from the cytosol *via* dedicated transporters or regulated trafficking at membrane contact sites with other endomembrane compartments. Stored triacylglycerols are then released from LDs, sites of neutral lipid storage enclosed by a phospholipid monolayer, which are then hydrolyzed into fatty acids by lipases and converted to fatty acyl-CoA by fatty acyl-CoA synthetase. Fatty acyl-CoA molecules must be converted into fatty acyl carnitine by the carnitine palmitoyltransferase 1 (CPT1) on the OMM for uptake by dedicated trans-membrane transport by carnitine acylcarnitine translocases. Within the mitochondrial matrix, carnitine palmitoyltransferase 2 (CPT2) converts the fatty acyl carnitine back into fatty acyl-CoA. These fatty acids are then broken down into two carbon acetyl-CoA units that are shuttled into the TCA cycle for ATP production (7, 8, 9).

Mitochondria are sites of lipid catabolism and key players in the *de novo* synthesis of long chain fatty acids, complementary to dietary lipid intake of short and long chain fatty acids (9, 10). Lipogenesis requires both cytoplasmic and mitochondrial enzymes, all of which are encoded in the nuclear genome that results in the end product palmitic acid (PA) (11, 12). ER-resident enzymes facilitate the elongation and desaturation of PA (13). The synthesized fatty acids can then be used to form phospholipids that integrate into mitochondrial membranes, which are needed for mitochondrial membrane growth and division. While fatty acid synthesis primarily occurs in the cytosol, one mitochondrial fatty acid synthase has been identified in mammals to create at least two products: octanoate eight carbon chain saturated fat and a long acyl chain of fourteen to sixteen carbons (14, 15). Loss of this mitochondrial pathway can result in reduced protein lipoylation and reduced ETC complex assembly, directly linking mitochondrial lipid synthesis to ATP production (15).

### Mitochondrial lipid metabolism is tuned at the micron scale by membrane interactions with other organelles

Mitochondrial lipid metabolism is not an isolated process; other lipid metabolizing organelles, such as the ER and LDs, directly interact with mitochondria for the transfer, anabolism, and catabolism of fatty acids that are transported into the cell. Fatty acids can be accessed for mitochondrial beta-oxidation directly from the cytosol or from LDs through contact sites. Therefore, study of mitochondria, ER, and LDs as a system instead of as isolated components can provide critical insights into mitochondrial lipid metabolism.

#### Mitochondria and lipid droplet interactions

The role of mitochondria in the oxidation or synthesis of lipids is directed by their contact with LDs, and these interactions are dictated by the nutritional status of the cell along an axis from starvation to lipid excess. In 2018, Benador *et al.* reported the identification of two subpopulations of mitochondria with differing lipid metabolizing paradigms: peridroplet mitochondria (PDM) (Fig. 2*B*) and cytoplasmic mitochondria (CM) (Fig. 2*A*). PDM formed close membrane contact sites with LDs. They found that PDM typically remained adjoined to LDs during density gradient centrifugation. PDM have higher respiratory capacity as measured by oxygen consumption rate, ATP production rate, pyruvate oxidation capacity, as well as OXPHOS protein levels, such as cytochrome c oxidase and ATP synthase, with lower fatty acid oxidation capacity, than CM. These findings led the authors to conclude that PDM function in triglyceride synthesis and transfer to LDs for storage. Investigation by electron microscopy revealed that PDM tend to be longer and tubular than CM, suggesting a link to mitochondrial dynamics. In live laser scanning confocal microscopy, PDM displayed lower rates of fission and fusion over 1 h, measured by a mitochondrial matrix–targeted photoactivatable green fluorescent protein and decreased motility, in time lapse imaging (16, 17). Such heterogeneity among mitochondrial subtypes may allow metabolic flexibility at the cellular and subcellular levels. For example, the relative proportions of PDM and CM mitochondria may be adjusted to meet cell type–specific needs or individual organelles may be trafficked to subcellular target locations to affect local lipid synthesis or breakdown.

**Figure 2:**
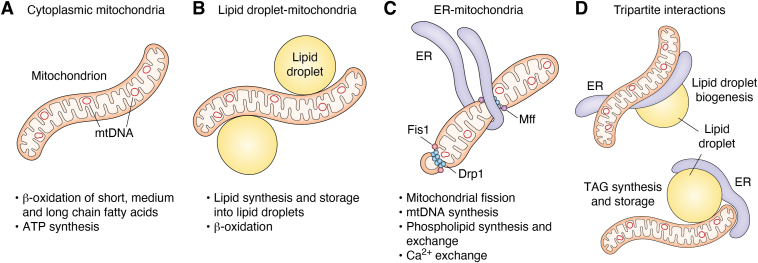
**Functional organization of mitochondrial contact sites.***A*, In the context of lipid metabolism, cytoplasmic mitochondria breaks down cytosolic fatty acids during β-oxidation to produce acetyl-CoA molecules that are channeled into the TCA cycle for respiration. *B*, Mitochondrial-lipid droplet interactions may function in both lipid synthesis, for lipid storage in droplets, as well as oxidation for energy production, depending on the state of the cell. *C*, ER–mitochondrial interactions are necessary for mitochondrial fission, and ER-mitochondrial division sites mark locations for mtDNA synthesis and segregation into daughter mitochondria. Drp1 and mid-zone receptor Mff facilitates the process of mitochondrial division at ER-mitochondria contact sites, and smaller division sites can occur at the periphery of the mitochondria without the need for ER-mitochondrial contact, facilitated by Drp1 and peripheral receptor Fis1. *D*, Tripartite interactions can include both a conformation where the ER is “sandwiched” between the mitochondria and budding lipid droplet during lipid droplet biogenesis (top) and a conformation of the ER wrapping around the lipid droplet and mitochondria for TAG synthesis and storage. Drp1, Dynamin-related protein 1; Fis1, Fission Protein 1; Mff, mitochondrial fission factor; TAG, triacylglycerol.

Hepatic CM and PDM exhibit distinct proteomic profiles, in which CM associated with ER-mitochondrial contact sites were relatively enriched for proteins associated with fatty acid oxidation, as well as subunits of all five respiratory chain complexes. PDM were relatively enriched with proteins with molecular functions relevant to lipid synthesis and storage, which the authors inferred could promote adaptation to lipid-induced toxicity. CM and PDM functional distinctions can be observed during different nutrient states: starvation promotes fatty acid trafficking to CM for oxidation and energy production, while the fed state promotes fatty acid trafficking to PDM to be esterified and stored in LDs (18). In contrast to these findings, in myoblasts, mitochondria tethered to LD-demonstrated increased transport and oxidation of fatty acids by interactions with the OMM acyl-CoA synthetase protein Fatp4 during starvation (19). The extent to which LD–mitochondrial interactions are modulated by nutritional status *in vivo* has been studied in few model systems but the studies reveal intriguing parallels. In Wistar rats, a model for nonalcoholic fatty liver disease, PDM demonstrated higher beta-oxidation capacity and reduced OXPHOS capacity than CM in rats-fed *ad libitum*; however, in PDM isolated from high-fat diet (HFD)-fed rats, fatty acid oxidation is significantly reduced compared to PDM isolated from *ad libitum* fed rats, with no difference compared to HFD-isolated CM (20). These contrasting findings suggest that there are differing localization and functions of mitochondrial subpopulations in the context of lipid metabolism, but the factors that determine PDM *versus* CM function may be cell-type–dependent. Fully understanding the contextual differences that influence PDM *versus* CM is still yet to be analyzed, as well as whether these mitochondrial subpopulations differ in the their mtDNA genome mutations or abundances.

#### Mitochondria and ER interactions

ER–mitochondria interactions, also known as mitochondria-associated membranes, were identified as early as the 1950s *via* electron micrographs of intact endomembranes (21). ER and mitochondrial interactions are important for metabolite exchange, such as calcium (Ca^2+^), phospholipids, and cholesterol (22). Close contact between mitochondria and ER at mitochondria-associated membranes permits the transfer of phospholipid precursors to mitochondria essential to mitochondrial membrane integrity and the synthesis of mitochondrion-specific lipid species including cardiolipin (22, 23). Mitochondrial membranes are composed of phosphatidylserine, phosphatidylethanolamine, phosphatidylinositol, phosphatidylcholine, phosphatidylglycerol, phosphatidic acid, and cardiolipin. Most cellular phospholipid synthesis occurs in the ER, with partial synthesis of phosphatidylethanolamine and phosphatidylglycerol occurring in the IMM (23, 24, 25, 26, 27). Disruption of phospholipid composition in either mitochondrial bilayer perturbs mitochondrial fusion and fission dynamics, though the molecular mechanisms remain unclear (28, 29, 30, 31). In contrast, the synthesis of long chain acyl-coenzyme A (LCACA), a substrate of the lipid synthesis pathway, provides a direct connection between lipid metabolism and mitochondrial division. LCACA interacts with the mitochondrial dynamic proteins MIEF1 and MIEF2, which serve as recruiting proteins for the mitochondrial fission mediator dynamin-related protein 1 (Drp1). Increased synthesis of LCACA by long chain fatty acid supplementation, such as with oleic acid, stimulates MIEF1/2 oligomerization and Drp1 recruitment for mitochondrial fission (32). Fusion and fission are necessary for the even distribution of metabolites and mtDNA between mitochondria. Additional molecular studies that detail how lipid metabolism can regulate mitochondrial dynamics could reveal new insight in the segregation and localization of mitochondrial genomes in coordination with membrane growth.

ER-mitochondria contact sites serve as platforms for mitochondrial fission during growth as well as mtDNA synthesis (Fig. 2*C*) (3, 33, 34, 35). Mitochondrial DNA replication and segregation are spatiotemporally coupled to mitochondrial division; thus, mitochondrial membrane growth *via* lipid synthesis may be spatially linked to mtDNA maintenance; further studies using both mtDNA nucleoid and lipid synthesis markers are needed. The composition and stability of the mitochondrial membranes affects mtDNA integrity, as mtDNA nucleoids are localized near the IMM in membrane domains putatively enriched for cardiolipin and cholesterol (36). Coupling of mitochondrial membrane growth with division and mtDNA synthesis serves to scale mtDNA nucleoid content to cellular mitochondrial mass (3, 33).

While the repertoire of membrane tethers and transport proteins needed for lipid exchange in mammalian cells is still expanding, one possible protein identified is Mitogaurdin-2 (MIGA2). MIGA2 is an OMM protein that has an FFAT motif (two phenylalanines in an acidic tract), an acidic amino acid region commonly found in lipid-transferring proteins, that interacts with the ER transmembrane VAMP-associated protein B. Additionally, MIGA2 has an LD-targeting domain that contains a hydrophobic pocket where lipids, particularly phospholipids, can be “captured” in this pocket for transport between the ER, mitochondria, and LDs (37, 38). In addition to phospholipid transfer, MIGA2 promotes ER–mitochondrial interactions for lipogenesis and storage of triglycerides in LDs (39). These findings suggest the possible formation of three way contact between ER, mitochondria, and LDs for the transferring of lipids between the organelles.

#### Mitochondria, LD, and ER tripartite interactions

Three-way contact sites between ER, mitochondria, and LDs, termed tripartite contacts, are a fairly new area of research in the lipid metabolism field. Two proteins that have been identified to bring together ER-mitochondria-LD contacts are the oxysterol binding protein and related proteins (ORP). ORP5 and ORP8 are lipid-binding and transfer proteins that associate with ER membranes *via* a C-terminal transmembrane segment; ORP5/ORP8 are locally enriched at ER-mitochondrial contact sites (40). As previously discussed, LD biogenesis occurs at the ER; ER-transmembrane acyltransferases synthesize triacylglycerols, which are transferred between the leaflets of the ER membrane bilayer to form a nascent LD. The ER membrane protein seipin will oligomerize to bud off the LD, allowing the LD to fully detach (41). Interestingly, ORP5 has been reported as necessary to the recruitment of seipin to ER-mitochondria-LD contacts and loss of ORP5/8-compromised LD biogenesis (42). Another study showing ER–mitochondria–LD tripartite interactions’ contribution to lipid homeostasis used serial block face scanning electron microscopy, where images displayed ER “sandwiched’ in between mitochondria and LD; these tripartite interactions increased during the fasting state (Fig. 2*D*) (18).

An exciting avenue of future research will be to develop and apply tools to visualize, characterize, and manipulate defined subpopulations of membrane contacts *ex vivo* and *in vivo*. Profiling variability among membrane contact site populations at the cellular scale can reveal how the number, cytoplasmic distribution, and/or temporal persistence of contact site classes contribute to organelle remodeling, for example, to tune the pathways of lipid homeostasis to mitochondrial membrane growth and mtDNA replication cues (43, 44).

### Lipotoxicity impairs mitochondrial function

#### Defining lipotoxicity

Lipotoxicity is often defined as the accumulation of fatty acids in nonadipose metabolic tissue that causes cellular dysfunction and cell death (45, 46) (Fig. 3*A*). Entry of fatty acid into peripheral tissues, particularly saturated fatty acids, such as PA, is linked to tissue damage identified by organ dysfunction, increased inflammation, and cellular death (47). Many studies of lipotoxicity have employed PA in cell culture or HFD in murine models to induce cellular or tissue damage due to these effects, and studying the effects of other saturated fatty acids as potential inducers of lipotoxicity is yet to be systemically explored (48, 49, 50). Such tissue damage of peripheral organs by lipotoxicity contributes to several metabolic disorders with mitochondrial involvement, including cardiomyopathy, kidney disease, non-alcoholic fatty liver disease, and type 2 diabetes mellitus (47, 51). Lipotoxicity was first defined in a study which found that elevated triacylglycerol levels in the pancreatic islets of Zucker diabetic fatty rats correlated with an increase in plasma free fatty acids (FFAs) and plasma glucose levels. Moreover, the culture of rat pancreatic islets with FFAs caused a reduction in glucose-stimulated insulin secretion, indicating impaired β-cell function in an excess lipid environment and showing the close relationship between glucose and lipid metabolism at the cellular level (52). The connection between lipotoxicity and metabolic disease has primarily been explored in murine models fed with an HFD with observations of dysregulated glucose and lipid homeostasis, but the extent to which murine models recapitulate human disease states is still poorly understood (50, 53, 54).

**Figure 3:**
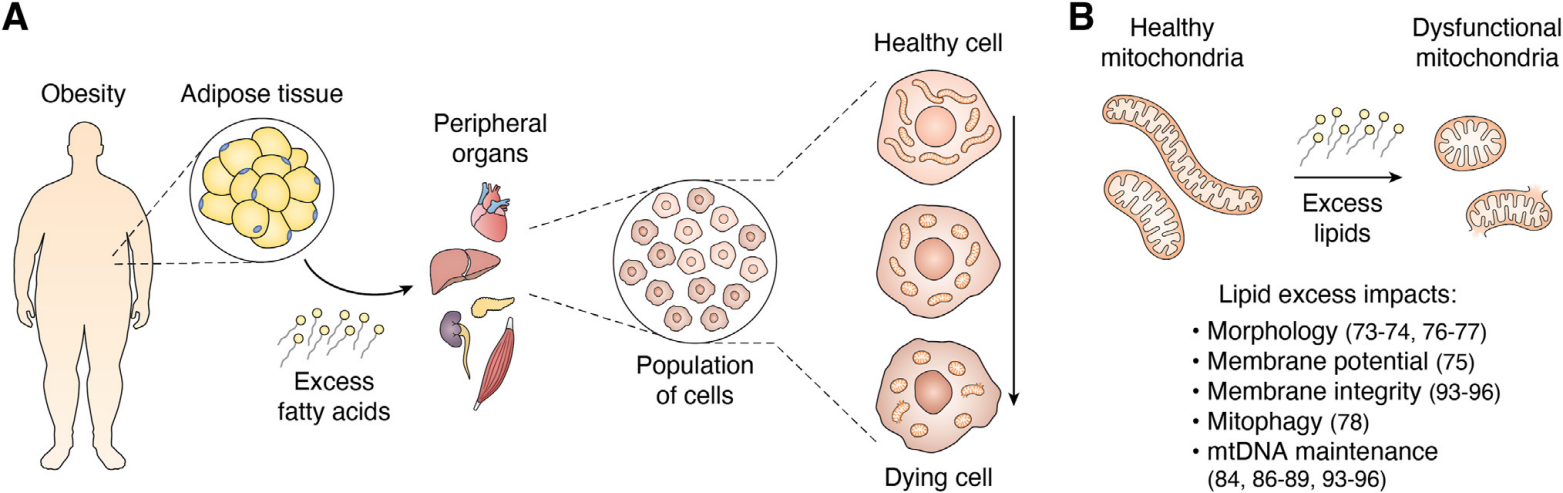
**Lipotoxicity and mitochondrial dysfunction across biological scales.***A*, During obesity, there is an accumulation of excess fat in adipose tissue. Fatty acids released into circulation from adipose tissue can enter into peripheral organs, such as the (heart, liver, skeletal muscle, kidney, and pancreas). Periphery tissue damage resulting from fat accumulation is termed lipotoxicity, where excess FAs cause cellular dysfunction. A population of cells in periphery tissue can gradually transition from healthy to dying during chronic excess lipid exposure. *B*, Mitochondria can become dysfunctional during these conditions due to changes in their morphology and function, as well as the maintenance of their genomes.

At the cellular level, lipotoxicity can cause diacylglycerol and ceramide accumulation, ER stress, activation of inflammatory pathways, increased autophagy, mitochondrial dysfunction *via* oxidative stress, and mtDNA damage, such as DNA lesions, which are all signs of cellular dysfunction and the likely impending commitment to cell death (45, 55, 56, 57). The interplay of these effects or whether one class of impairment supersedes others remains poorly understood. As described, mitochondria coordinate lipid metabolism at contact sites with other organelles, and lipotoxicity could plausibly impair these interactions (20, 58, 59). More work is needed to parse the contribution of lipid excess to mitochondrial contact site remodeling and declining cellular health to create mechanistic models of mitochondrial responses to lipotoxicity.

#### Lipotoxicity induces cell death

PA is a saturated, long chain fatty acid produced during mammalian fatty acid synthesis and is a commonly found saturated fatty acid in the diet (12). Cells fed excess PA *in vitro* exhibit reduced cell viability, specifically through activation of apoptosis, which is an extrinsic or intrinsic programed cell death (60, 61, 62, 63). Other forms of cell death are induced by excess FFA in specific contexts, including pyroptosis and necroptosis (64, 65, 66, 67, 68). Ferroptosis, an iron-dependent form of programmed cell death, produces lipid-free radicals through the peroxidation of polyunsaturated fatty acids. The accumulation of these lipid peroxides leads to lipid and protein damage (69, 70, 71). The direct or indirect role of mitochondria in these nonapoptotic cell death pathways is as yet unclear.

#### Lipotoxicity and mitochondrial dysfunction

Cells exposed to a lipotoxic environment demonstrate features of mitochondrial dysfunction, including mitochondrial network fragmentation and reduced mitochondrial membrane potential (Fig. 3*B*) (72, 73, 74). Remodeled mitochondrial morphology is associated with changes in nutrient uptake and catabolism in metabolic disease (75). Consistently, pancreatic islets isolated from Zucker diabetic fatty rats exhibited shortened mitochondria with swollen architecture suggestive of cristae maintenance defects (76). Genetic induction of mitochondrial fragmentation can, in some cell types, recapitulate functions of increased fatty acid oxidation, suggesting that mitochondrial form plays a physiological, cell type–specific role in lipid catabolism (75). These findings raise the possibility that the impact of lipotoxicity on mitochondrial function in cells and tissues may be modified by the mitochondrial network pre-lipotoxic insult.

Interestingly, several groups have shown that excess of either saturated or unsaturated fatty acids induces autophagy in cellular models, contributing to elevated mitochondrial turnover in lipotoxic conditions. While PA treatment upregulates canonical autophagy dependent on adenosine monophosphate-activated protein kinase, oleate treatment stimulates a non-canonical autophagy pathway that relies on Golgi signaling (Fig. 3*B*) (77). Given that lipid mobilization is a signal of cellular stress and low glucose availability, induction of autophagy by fatty acid excess may serve a quality control purpose to suppress lipotoxic damage to cells. Whether the strength of autophagic induction differs in starvation *versus* nutrient excess or obesity is yet to be discovered. There is some evidence to support that autophagy regulates mtDNA copy number as well as the presence of mutated mtDNA; surprisingly, mechanistic studies of mtDNA maintenance and autophagy during starvation or nutrient excess in *in vivo* animal models lag behind, given that the influence of dietary lipid intake on mitochondrial membrane composition could provide a means of perturbing both mtDNA nucleoid homeostasis and cristae morphology (78, 79, 80).

### Lipid metabolism and mitochondrial DNA integrity are interdependent

#### mtDNA damage during oxidative stress

Production of reactive oxygen species is prominent in mitochondria due to reactions of electrons at ETC complexes with oxygen molecules, producing superoxide anions and hydroxyl radicals (81, 82). Free radicals are molecular species containing atoms with unpaired electrons that can react with nonradical species, including macromolecules—lipids, proteins, nucleic acids, and carbohydrates (81). While the majority of mtDNA mutations arise *via* replication errors, mitochondrial nucleoids are located near the IMM and cristae, the location of respiration complexes, and therefore ROS generated by OXPHOS complexes has been proposed to cause oxidative damage to nearby mtDNA (57, 83) (Fig. 4.3).

**Figure 4:**
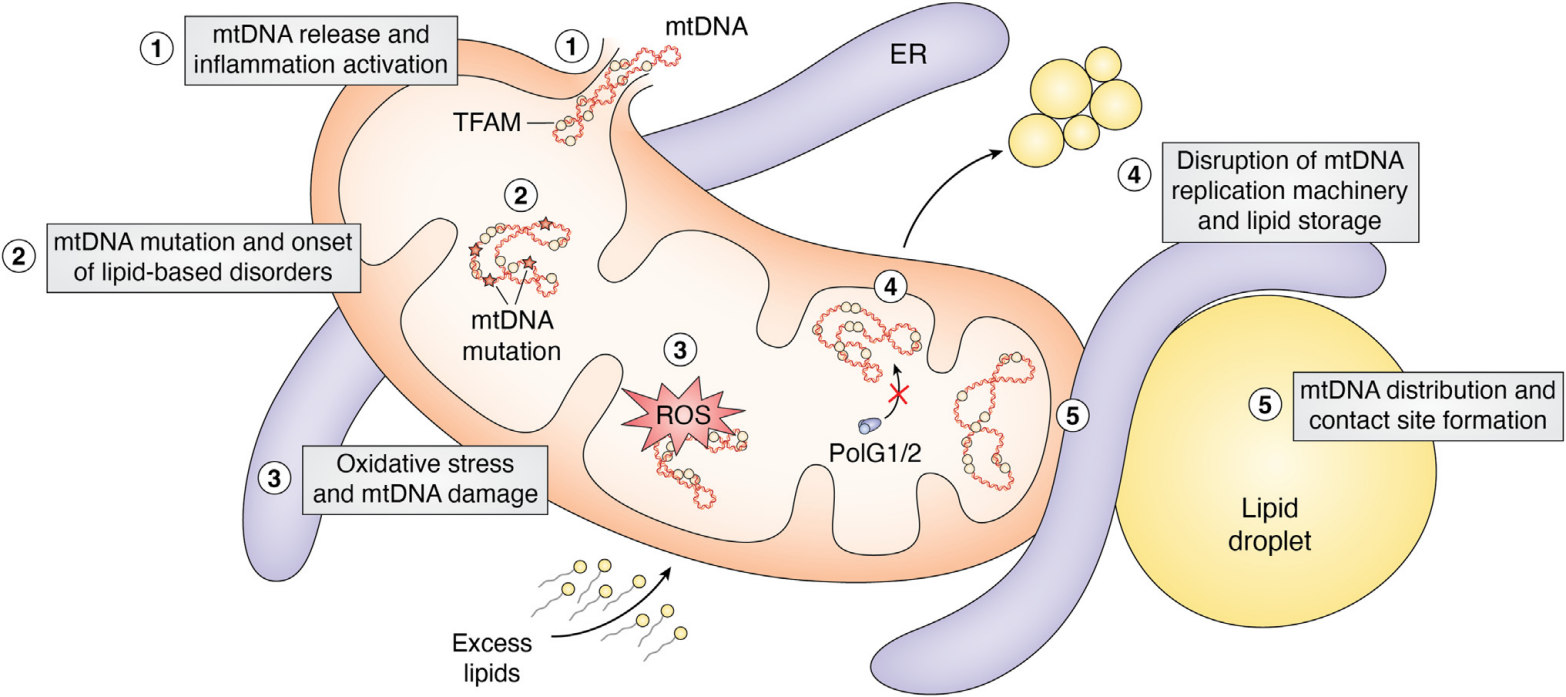
**Lipid excess is linked to mtDNA maintenance.** Displayed are five ways that an excess lipid environment could impact mtDNA maintenance: (1) mtDNA release from the mitochondrial matrix into the cytosol may activate inflammatory pathways; (2) mtDNA mutations can cause metabolic, and more specifically, lipid-based disorders; (3) oxidative stress by reactive oxygen species (ROS) can result in mtDNA damage, such as DNA lesions; (4) disruption of mtDNA replication machinery can lead to perturbed lipid storage; (5) mtDNA distribution across the mitochondrion and in relation to contact sites, such as tripartite contact sites, may affect lipid metabolism.

In the lipotoxic context, mouse INS-1 cells treated with excess FFAs high in PA had activated nitric oxide synthase and increased nitric oxide levels. Elevated nitric oxide levels damaged mtDNA by generating oxide products, such as nitrous anhydride (N_2_O_3_), that created mtDNA lesions through deamination reactions (82). Rachek *et al.* demonstrated a similar result upon treatment with palmitate with increased superoxide and nitrite levels. This increase in free radicals correlated with increased mtDNA breaks, as well as decreased cellular ATP levels and cell viability (84). Whether mtDNA lesions may be repairable is controversial. Human 8-oxoguanine DNA glycosylase (OGG1) is a DNA base excision repair enzyme. While the mechanism of mitochondrial base excision repair pathways are not well understood, INS-1 cells overexpressing a transfected mitochondrial targeting sequence vector encoding OGG1 (MTS-OGG1) showed reduced mtDNA damage and apoptosis upon FFA treatment (85). The relationship between mtDNA damage and lipid metabolism can also be observed at the physiological level: It has been demonstrated that oxidative damage of mtDNA is linked with increased adiposity and obesity onset in mice (Fig. 3*B*) (86, 87). Following investigations show that overexpression of OGG1 is a means to regulate adiposity in mice, with OGG1 KO displaying increased fat accumulation (88). Alternative ways to prevent mtDNA damage during lipotoxic-induced oxidative stress remain elusive.

#### mtDNA release and activated inflammatory pathways

Mitochondrial DNA can serve as a signaling molecule during cellular stress for the activation of immune pathways such as the inflammasome, toll-like receptor 9, and cGAS-STING (89). Immune activation occurs downstream of mtDNA efflux from the mitochondrial matrix into the cytosol. The exact mechanism by which mtDNA becomes accessible to the cytosol is not known, but in recent years, the release of mitochondrial nucleic acid into the cytoplasm has been reported during a variety of cellular stress states, typically imminent to cell death. In McArthur *et al.*, mouse embryonic fibroblasts that were treated with an apoptotic-inducing drug led to compromised integrity of the mitochondrial membrane, inducing permeabilization of the mitochondria by the oligomerization of BAK and BAX proteins, which form pores on the outer membrane. They state that the herniating IMM exposes the matrix-housed mtDNA to the innate immune sensor, cGAS, in the cytosol, activating the cGAS–STING pathway (90). While the full release of mtDNA into the cytosol was not indicated in this report, the findings were an advance in visualizing mtDNA release through a combination of super-resolution live imaging and electron microscopy. Further investigation is needed to determine the status of the cell and the factors involved that influences its release, as well as if there is a way to prevent mtDNA release for cell death prevention. Live cell imaging to visualize the full release of mtDNA into the cytosol and its interaction with cGAS-STING could enhance our understanding of mtDNAs role in cell death and innate immunity.

One route by which PA treatment may reduce cell viability in culture is mtDNA release from permeabilized mitochondria and the subsequent activation of the innate immune response (Fig. 4.1) (91, 92, 93). Another report showed that mtDNA release and activation of toll-like receptor 9, another pro-inflammatory marker, led to nonalcoholic steatohepatitis in mice (Fig. 3*B*) (94). mtDNA release may not solely be an apoptotic event, as it may also occur during other forms of cell death, such as pyroptosis or necroptosis (65, 95).

#### Perturbed lipid storage in primary mitochondrial disease

How does the interrelationship between lipid homeostasis and mtDNA-dependent mitochondrial function contribute to disease? A dedicated mitochondrial replisome consisting exclusively of nuclear-encoded proteins replicates mitochondrial genomes (83). MtDNA replication defects cause mitochondrial myopathy, peripheral neuropathy, and a starvation-like response in mice even while in a normal nutritional state; this activates alternative energy production pathways, leading to reduced adipocyte size and reduced liver fat content (96). Several studies have identified LD expansion as a signature of mtDNA depletion and/or deletion in mouse models (97). Transcriptomic analysis in a mouse model depleted of the mitochondrial helicase suggested progressive impairment of lipid storage; consistently, fluorescence microscopy revealed a dramatic expansion of LDs in astrocytes depleted of the mitochondrial helicase TWINKLE (98). In a DNA polymerase gamma mutant mouse model, lipidome remodeling was a key feature of metabolic disease progression and correlated with reduced triacylglycerols and hypoglycemia (99). Consistently, human mitochondrial disease patients with DNA polymerase gamma mutations exhibit enlarged LDs in muscle and in hepatocytes (97, 100). Taken together, these studies indicate that mtDNA replication defects are linked with disrupted lipid homeostasis either directly or indirectly (Fig. 4.4).

#### mtDNA mutations and metabolic disease

Quality control of the polyploidy mitochondrial genome is necessary for overall mitochondrial and respiratory function, as mtDNA mutations cause myriad heritable musculoskeletal and neurodegenerative disorders and are also closely linked to other metabolic diseases including diabetes and glucose intolerance (101, 102). Maternally Inherited Diabetes and Deafness is caused by an mtDNA mutation in the mt-tRNA^Leu^ gene, which leads to decreased OXPHOS function and ATP production. This, ultimately, reduces insulin secretion and promotes beta cell apoptosis. In addition to hyperglycemia and hearing loss, other clinical features include macular dystrophy, cardiac failure, and short stature (103, 104, 105). Kearns-Sayre is an mtDNA-mutation disorder identified by an mtDNA deletion that can range from 2000 to 7000 bp (106, 107). While the key features of this disease are ophthalmoplegia, bilateral pigmentary retinopathy, cardiomyopathy, and cognitive impairment, there are cases where individuals with this disorder also develop diabetes, requiring insulin injection for hyperglycemia (108, 109, 110). There is a clear connection between glucose and lipid homeostasis in type 2 diabetes mellitus, which raises the possibility that mtDNA-linked diabetes may be associated with systemic lipid dysregulation (Fig. 4.2).

Clinical manifestations of mtDNA-disease indicate that mtDNA integrity is closely tied to lipid homeostasis, but cellular models form many of these diseases remain to be developed. Nonalcoholic fatty liver disease, which is commonly associated with obesity and an HFD, can progress into steatohepatitis (NASH) or liver failure. A mutation in the mt-CYB gene, encoding for the OXPHOS protein cytochrome b, was associated with NAFLD progression into NASH (111). Another lipid-based disease caused by mtDNA mutation is lipomatosis, an abnormal overgrowth of adipose tissue in typically lean body regions. Mitochondrial mt-tRNA^Lys^ mutations are linked to lipomatosis (112, 113, 114, 115, 116). Another mutation in the mt-tRNA^Leu^ gene was identified to cause hyperglycemia associated with lipoma development and polyneuropathy, which the authors speculate could be a possible link between mitochondrial diabetes and disrupted systemic lipid metabolism (117). While certain mitochondrial DNA alleles are correlated with higher rates of obesity, mechanistic studies that could identify mtDNA variants associated with obesity in otherwise isogenic animal models are needed (118, 119, 120, 121, 122, 123, 124).

## Perspective

Mitochondrial metabolism is central to cellular homeostasis, yet these organelles do not perform their myriad bioenergetic, biosynthetic, and catabolic functions in isolation. Mitochondria must interact with other cellular organelles and cytosolic metabolite signaling pathways for efficient lipogenesis or fatty acid oxidation. Studies in the model invertebrates such as *Caenorhabditis elegans* and *Drosophila melanogaster* will be useful in this regard because they provide a foundation for understanding how mitochondrial form and function are linked in homeostasis and can reveal fundamental cellular mechanisms shared among animals. Future effort to perform *in vivo* imaging of live animal models will be essential to dissect this relationship across different tissues and cell types. New sensors and probes that can reveal the genesis and fates of lipid species and their contributions to lipotoxic phenotypes *via* dynamic tracking over time in live cells and animals are needed.

Looking forward, discovering methods to repair or reverse lipotoxic insult to mitochondria may lead to treatments for damage tissues. An endogenous response that could improve lipotoxic conditions of the cell is the sequestration and neutralization of excess lipids into LDs for storage. A proposed concept is that LD sequestration and neutralization of the cytosolic FFA pool and toxic lipid by-products, such as ceramides and diacylglycerols, could reduce altered lipid composition of the ER membrane, preventing activation of ER stress pathways (41). Mitochondrial fatty acid overload could also be modulated *via* the inhibition of import proteins (125). Combining sequestration of fatty acids into LDs and partial inhibition of fatty acid import into mitochondria during high-fat intake could mitigate cellular lipotoxicity. A nutritional means to mitigate lipotoxic impairment of mitochondria in cells could be the use of mono- and poly-unsaturated fatty acids, which has been reported to ameliorate the effects of PA treatments and HFDs (126, 127, 128, 129, 130, 131, 132, 133). Finally, inhibiting mitochondrial transcription led to improved weight management and reduced liver fat content in HFD-fed mice, supporting a connection between mtDNA-encoded gene expression and lipid metabolism at the organismal level (134). Further investigation of how modulation of the mitochondrial central dogma may integrate with lipid metabolism could lead to advancements in drug treatments for both lipid-based disorders and primary mitochondrial disease.

While the harmful effects of excess fatty acids on mitochondria are coming into focus, how mitochondrial and cellular stress responses to lipid dysregulation are induced and regulated is less clear. Even more so, it is unclear if links between lipid dysregulation and mtDNA maintenance are direct or indirect. Potentially fruitful avenues of future research include the following: (1) dissecting the role of membrane contact sites on mtDNA maintenance during excess lipid exposure (Fig. 4.5), (2) determining whether altered lipid composition of the mitochondrial membrane and impaired membrane integrity affects maintenance of the mitochondrial genome, (3) investigating if induction of autophagy due to lipid excess could lead to breakdown of mitochondria that may indirectly impact mtDNA levels and localization in the cell. There is strong evidence to support that mtDNA replication and integrity are mechanistically linked to cellular lipid homeostasis, but how mtDNA maintenance may be impacted during compromised nutritional states, such as starvation and lipid excess, is poorly understood. This is particularly in the context of postmitotic peripheral tissues. Unraveling these mysteries may uncover novel therapeutic approaches to primary mitochondrial disease, metabolic syndromes, and even obesity.

## Open questions

• How heterogeneous are intracellular mitochondrial populations in terms of metabolic function? How does this heterogeneity vary among cell types?
• Could heterogeneity in mitochondrial disease manifestations between tissues be attributed to cell type–specific interplay between mitochondrial roles in lipid homeostasis and energy production?
• Is mtDNA copy number impacted by lipotoxicity, for example, *via* uncoupling of mitochondrial membrane growth from mtDNA replication?
• Is mtDNA release an obligate component of cell death downstream of compromised mitochondrial membrane integrity or can it be triggered independently?
• Can lipotoxic insult to mitochondria be repaired or reversed, and can overall cellular health be restored after lipotoxic damage?

## Conflict of interest

The authors declare that they have no conflicts of interest with the contents of this article.
